# Lifetime congenital isolated GH deficiency does not protect from the development of diabetes

**DOI:** 10.1530/EC-13-0014

**Published:** 2013-06-15

**Authors:** Taísa A R Vicente, Ívina E S Rocha, Roberto Salvatori, Carla R P Oliveira, Rossana M C Pereira, Anita H O Souza, Viviane C Campos, Elenilde G Santos, Rachel D C Araújo Diniz, Eugênia H O Valença, Carlos C Epitácio-Pereira, Mario C P Oliveira, Andrea Mari, Manuel H Aguiar-Oliveira

**Affiliations:** Division of Endocrinology Federal University of Sergipe Aracaju, Sergipe, 49060-100 Brazil; 1 Division of Endocrinology The Johns Hopkins University School of Medicine 1830 East Monument Street, Suite #333, Baltimore, Maryland, 21287 USA; 2 National Research Council Padova, 35127 Italy

**Keywords:** insulin sensitivity, β-cell function, diabetes, GH deficiency

## Abstract

**Objectives:**

Adult subjects with untreated, lifetime, isolated GH deficiency (IGHD) due to a homozygous GHRH receptor gene mutation (MUT/MUT) residing in Itabaianinha, Brazil, present with lower BMI, higher prevalence of impaired glucose tolerance (IGT), increased insulin sensitivity (IS), and reduced β-cell function (βCF) when compared with non-BMI-matched homozygous normal controls. However, the prevalence of diabetes mellitus (DM) in this cohort is unknown. Comparing their IS and βCF with BMI-matched individuals heterozygous for the same mutation (MUT/N) may be useful to elucidate the role of the GH–IGF1 axis in IS and βCF. The purposes of this work were to verify the prevalence of IGT and DM in adult MUT/MUT subjects from this kindred and to compare IS and βCF in MUT/MUT and MUT/N.

**Design:**

Cross-sectional study.

**Methods:**

We studied most (51) of the living IGHD adults of this kindred who are GH naive. The oral glucose tolerance test (OGTT) could be performed in 34 subjects, fasting glucose was measured in 15, while two had a previous diagnosis of DM. The OGTT results of 24 MUT/MUT subjects were compared with those of 25 BMI-matched MUT/N subjects. IS was assessed by homeostatic model assessment of insulin resistance (HOMA–IR), quantitative IS check index, and oral glucose IS index for 2 and 3 h. βCF was assayed by HOMA-β, insulinogenic index, and the area under the curve of insulin:glucose ratio.

**Results:**

The prevalence of DM and IGT in IGHD was 15.68 and 38.23% respectively. IS was increased and βCF was reduced in MUT/MUT in comparison with MUT/N.

**Conclusions:**

Lifetime, untreated IGHD increases IS, impairs βCF, and does not provide protection from diabetes.

## Introduction

GH regulates β-cell function (βCF) and insulin sensitivity (IS) both directly and via complex interactions with its principal mediator, insulin-like growth factor 1 (IGF1) (1). While GH reduces IS, IGF1 increases it. The hyperglycemic action of GH occurs mainly in the liver, and the hypoglycemic action of IGF1 occurs mainly in muscle (2). At least in animal models, IGF1 also has an important role in the development of β-cell mass (3, 4, 5). Genetic forms of GH resistance or GH deficiency (GHD) may be useful to clarify the role of the GH–IGF1 axis in regulating insulin secretion and action in humans.

In contrast to obese individuals, who present with reduced IS, GH-resistant (Laron syndrome) dwarfs present with normal IS – as measured by homeostatic model assessment of insulin resistance (HOMA–IR) – despite increased fat mass percentage (6). In these subjects, βCF – assessed by the insulin response during an oral glucose tolerance test (OGTT) – also seems to be impaired, especially in subjects older than 23 years, with a case of diabetes mellitus (DM) diagnosed in a 38-year-old patient due to βCF exhaustion (7). However, recently, in a large Ecuadorian study of kindred with Laron syndrome, none of the GH-resistant individuals self-reported to have DM (8). This has attracted the attention of scientific and lay media, as has been interpreted as showing that in the absence of GH action, DM does not occur.

We have previously described a large extended kindred with congenital isolated GHD (IGHD) due to the c.57+1G>A mutation in the GHRH receptor (GHRHR) gene (*GHRHR*), who reside in Itabaianinha county in northeast Brazil (9). Individuals homozygous for this mutation (MUT/MUT) have very low serum GH and IGF1 levels (10), resulting in severe proportionate dwarfism. Despite several cardiovascular risk factors (11), they exhibit normal longevity (12) and no evidence of premature atherosclerosis (13). We have recently studied 24 IGHD subjects and found that, despite an increase in percentage fat mass they do not have insulin resistance, but have impaired βCF and a higher prevalence of impaired glucose tolerance (IGT) when compared with homozygous (N/N) normal controls (14). One important limitation of our previous report was that the MUT/MUT and N/N groups were not matched for BMI. It is difficult to match MUT/MUT with N/N individuals, as reduced BMI is a hallmark of congenital GHD. However, subjects who are heterozygous for the mutation (MUT/N) have a partial phenotype. Despite normal stature and serum IGF1 levels, they have reduced lean body mass and a similar BMI compared with MUT/MUT subjects (15). This renders the MUT/N group an ideal one to compare with the MUT/MUT group.

The purposes of this work were i) to extend the study of prevalence of impaired fasting glucose (IFG) and DM to all the adult IGHD subjects from Itabaianinha and ii) to compare the prevalence of IGT and the degree of IS and βCF in MUT/MUT and MUT/N subjects.

## Materials and methods

### Design

This was a cross-sectional study performed in Itabaianinha county in the northeastern Brazilian state of Sergipe. Subjects were recruited by an advertisement, placed in the local dwarfs' association building, and by word of mouth. Two groups were enrolled: MUT/MUT (IGHD homozygous individuals for the *GHRHR* c.57+1G>A mutation) and MUT/N (heterozygous for the same mutation living in the same community). Both the Federal University of Sergipe and the Johns Hopkins University Institutional Boards approved this study. All subjects signed a written informed consent. A two-step protocol was performed: the first epidemiological and the second physiological.

#### Experiment 1

We have identified 52 living untreated adult MUT/MUT individuals, 26 females, age 45.08 (20.03) years, range 18–97 years. Two of them had a previous diagnosis of DM (one on metformin and the other on metformin and glimepiride) and did not undergo OGTT. Depending on logistics and subjects' availability, some had only fasting glucose, some partial, and some complete OGTT. One individual declined to participate. We measured fasting glucose in 15 individuals, performed OGTT with glucose measurements at 0 and 120 min in 10 individuals, and complete OGTT (with glucose and insulin measurements at 0, 30, 60, 90, 120, and 180 min) in 24 additional individuals, for a total of 51 subjects. Data of this experiment are descriptive and were compared with Brazilian epidemiological studies.

#### Experiment 2

The results of previously reported OGTT performed in 24 MUT/MUT subjects (14) were compared with those of 25 age- and sex-matched MUT/N individuals. Glucose and insulin were measured at 0, 30, 60, 90, 120, and 180 min. Exclusion criteria were as follows: age <25 or more than 60 years, known history of DM, excessive alcohol use, chronic and systemic diseases, previous GH use, use of drugs that could impact IS and βCF, and fasting glucose ≥126 mg/dl.

### Laboratory methods and calculations

Glucose was measured by the enzymatic Trinder colorimetric test on the day of each test. Sera for insulin were frozen at −40 °C and assayed together. Insulin was measured by an immunofluorometric assay having a sensitivity of 0.5 μU/ml (PerkinElmer Life and Analytical Sciences, Turku, Finland), and intra-assay and interassay variabilities were 2.5 and 3% respectively. IS was assessed by three methods: i) HOMA–IR, measured by the formula: fasting serum insulin (μU/ml)×fasting plasma glucose (mmol/l)/22; ii) quantitative IS check index (QUICK), measured by the formula: 1/(log insulin (μU/ml)+log glucose (mg/dl)); and iii) oral glucose IS index for 2 h (OGIS2) and 3 h (OGIS3). βCF was estimated using the basal glucose and insulin values by HOMA-β (20×fasting insulin)/(fasting glucose (mmol/l)−3.5); the first rapid phase of insulin secretion, using the insulinogenic index (IGI) (insulin time 30−insulin time 0 (μU/ml))/(glucose time 30−glucose time 0 (mg/dl)); and the total glucose-adjusted insulin response during the OGTT, by the area under the curve of insulin:glucose ratio (AUC I (μU/ml):G (mg/dl)) (14).

### Anthropometric measurements

Height, body weight, waist circumference (W) at half the distance between the last rib and the superior margin of iliac crest, and hip circumference (H) at the level of femoral trochanters were measured. The W:H ratio was calculated, and BMI was obtained by the formula: weight (kg)/height (m^2^).

### Prediabetes and diabetes diagnosis

Current American Diabetes Association criteria were followed. Prediabetes was defined by the presence of either IFG (fasting glucose levels between 100 and 125 mg/dl) and/or IGT (2-h glucose level during OGTT between 140 and 199 mg/dl). DM was defined by a fasting glucose level ≥126 mg/dl or a 2-h glucose level during OGTT ≥200 mg/dl.

### Statistical analysis

Variables with normal and not-normal distribution were compared by *t*-test and Mann–Whitney *U* test respectively. Data of normal distribution are reported as mean (s.d.). Data with not-normal distribution (HOMA–IR, HOMA-β, IGI, QUICK-I, and BMI) are reported as median (interquartile range). Statistical analysis was performed using the Software SPSS/PC 15.0 (SPSS, Inc.). *P* values ≤0.05 were considered statistically significant.

## Results

### Experiment 1

The prevalence of IFG was 9.8% (five cases). The prevalence of DM was 15.7% (eight cases: two cases were previously diagnosed, two were diagnosed by fasting glucose ≥126 mg/dl, and four by 2-h post-glucose value ≥200 mg/dl). The prevalence of IGT in the MUT/MUT subject who underwent OGTT was 13/34 (38.2%). However, four of these subjects had IFG. Therefore, the prevalence of IGT in subjects with normal fasting glucose is 9/34 (26.4%).

### Experiment 2

**Figure 1:**
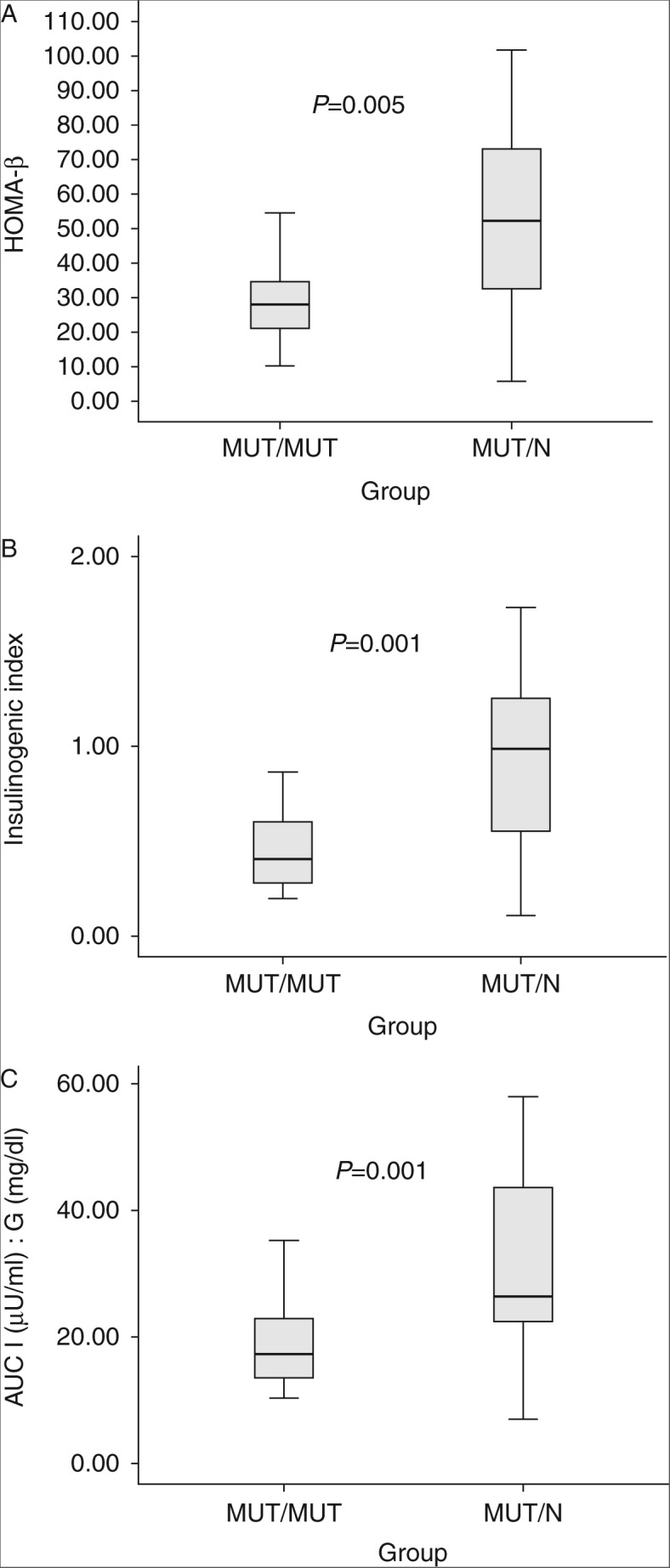
Boxplot of (A) HOMA-β, (B) IGI, and (C) AUC I (μU/ml):G (mg/dl) in 24 MUT/MUT subjects and 25 MUT/N subjects. The horizontal line in the middle of the rectangle is the median, the low margin of the rectangle is the 25th percentile, and the high margin of the rectangle is the 75th percentile.

As expected, height and weight were lower in MUT/MUT subjects than in MUT/N subjects. BMI was similar, while W:H ratio was higher in MUT/MUT subjects. HOMA–IR was lower and QUICKI was higher in MUT/MUT subjects than in MUT/N subjects (*P*=0.004 and *P*=0.005 respectively). OGIS2 had a trend toward being higher in MUT/MUT subjects than in MUT/N subjects (*P*=0.054), while OGIS3 was similar (Table 1). HOMA-β, IGI, and AUC I:G were lower in MUT/MUT subjects than in MUT/N subjects (*P*=0.005, *P*=0.001, and *P*=0.002 respectively; Table 1 and Fig. 1).

## Discussion

Recently, there has been great interest – both in the medical literature and in the lay press – about the report that adult dwarf individuals with GH resistance from a large Ecuadorian kindred do not have diabetes or cancer (8). Conversely, the prevalence of DM and IGT in our IGHD cohort was 15.7 and 38.2% respectively. Although the prevalence of DM we have observed was similar to that of DM in two population studies in Brazil (12.1 and 13.5%), the prevalence of IGT is even higher than that reported in these studies (5 and 7.7% respectively) (16, 17). The DM prevalence in our cohort may have been even higher if we had been able to perform an OGTT in all the 51 subjects. These findings prove that lifetime IGHD due to a *GHRHR* mutation – contrary to what has been reported in a large GH-resistant cohort – does not protect against development of DM.

It is possible that the metabolic consequences of GH resistance and GHD are different, particularly because patients with *GHRHR* mutations secrete a small but detectable amount of GH (18), which may contribute to a lower IS than the Laron model (where the GH effect is likely to be completely absent). It is conceivable that the lack of a GHRH effect *per se* may have additional consequences on glucose metabolism independent of GHD. It is also possible that the self-reporting approach may have underestimated the real prevalence of DM in the Ecuadorian subjects. Indeed, diabetes and its complications have been described in patients with Laron dwarfism (19, 20, 21). Accordingly, if we had relied only on self-reporting, we would have concluded that only two subjects (3.92%) have diabetes.

Interestingly, despite reduced glucose tolerance, our IGHD individuals present increased IS in comparison with a BMI-matched MUT/N group. These data are similar to the previous finding of increased IS in MUT/MUT in comparison to N/N. The limitation of the previous paper was that the MUT/MUT and N/N groups were not perfectly matched for BMI (14). Reduced BMI is a hallmark of congenital GHD (22) and GH resistance (6), due to small muscles and bones, and it is very difficult to match MUT/MUT with N/N individuals by BMI. In this study, we were fortunate to have a MUT/N group with a BMI similar to that of MUT/MUT group. Therefore, the present data confirm our previous conclusion that IGHD *per se*, despite increase in percentage fat mass, and contrary to what has been reported in adult-onset GHD (AOGHD), does not cause insulin resistance. We hypothesize that the difference in IS (increased in our IGHD individuals and reduced in AOGHD individuals) is probably caused by different degrees of GHD, and possibly influenced by co-morbidities, such as a lack of other pituitary hormones and associated therapies in AOGHD (23), and is not due to methodological issues. Indeed, the OGTT with simultaneous glucose and insulin measurement has an excellent correlation with the euglycemic–hyperinsulinemic clamp (24), the ‘gold standard’ method to assess IS, used in acquired AOGHD (25).

The increase of IS in the IGHD group may be caused by the marked reduction in GH levels and thereby lack of insulin antagonism. Additionally, increased muscle IS may be due to an increase in the molar ratio of total IGF to IGF binding protein type 3 (10), similar to what seen in healthy centenarians (26).

Such an increase in IS does not result in the prevention of diabetes. We speculate that this is due to the important effect of the GH–IGF1 axis on βCF, whose measures were reduced in MUT/MUT subjects. This reduction in βCF may be the decisive factor for the high prevalence of IGT in IGHD subjects. Conversely, as MUT/N subjects have normal IGF1 levels, they may have normal βCF.

Similar to our IGHD subjects, little mice (also carrying a homozygous *GHRHR* mutation like our subjects) (27) and mice with GH receptor gene ablation (GHRKO) (3) are hypersensitive to insulin, but have IGT due to a reduced number of pancreatic β cells and reduced insulin secretion, probably due to reduced pancreatic β-cell mass. Accordingly, *Igf1* gene overexpression in GHRKO animals restores β-cell mass, normalizing insulin production, and glucose tolerance (4).

In conclusion, severe congenital lifetime IGHD increases IS but impairs βCF and does not provide protection from DM, whose prevalence is similar to that of the general Brazilian population. Therefore, the dream of a ‘diabetes-free life’ is not present even in severe congenital IGHD.

## Figures and Tables

**Table 1:** Anthropometric and metabolic data in 24 MUT/MUT subjects and 25 MUT/N subjects involved in experiment 2. Data are expressed as mean (s.d.), except for HOMA–IR, HOMA-β, and IGI, which are expressed as median (interquartile range).

	**MUT/MUT**	**MUT/N**	***P***
Age (years)	39.25 (11.73)	40.08 (10.87)	0.798
Sex (males/females)	12/12	12/13	1.000
Height (cm)	128.21 (9.42)	160.66 (9.27)	<0.001
Weight (kg)	37.2 (5.95)	59.35 (10.26)	<0.001
BMI (kg/m^2^)	22.9 (4.68)	22.88 (2.79)	0.983
Waist (cm)	76.5 (9.99)	80.46 (7.72)	0.139
Hip (cm)	81.94 (8.01)	91.95 (7.9)	<0.001
W:H ratio	0.93 (0.09)	0.88 (0.09)	0.033
HOMA–IR	0.55 (0.49)	0.96 (0.57)	0.004
QUICKI	0.43 (0.04)	0.39 (0.04)	0.005
OGIS2	444.3 (49.6)	413.18 (59.74)	0.054
OGIS3	453.6 (62.14)	430.14 (72.2)	0.230
HOMA-β	28.1 (14.52)	52.24 (42.4)	0.005
IGI	0.4 (0.36)	0.99 (0.98)	0.001
AUC I (μU/ml):G (mg/dl)	0.22 (0.08)	0.36 (0.17)	0.001
